# Unveiling the therapeutic potential of miR‐146a: Targeting innate inflammation in atherosclerosis

**DOI:** 10.1111/jcmm.70121

**Published:** 2024-10-11

**Authors:** Azizah Puspitasari Ardinal, Alice Valeria Wiyono, Reza Ishak Estiko

**Affiliations:** ^1^ King's College London School of Cardiovascular and Metabolic Medicine London UK; ^2^ Bandung City Regional General Hospital Bandung Indonesia

**Keywords:** atherosclerosis, cardiovascular, inflammation, miR‐146a, plaque

## Abstract

Atherosclerosis is the foremost vascular disease, precipitating debilitating complications. Although therapeutic strategies have historically focused on reducing cholesterol deposition, recent insights emphasize the pivotal role of inflammation. Innate inflammation significantly contributes to plaque instability and rupture, underscoring the need for intervention across all disease stages. Numerous studies have highlighted the therapeutic potential of targeting innate immune pathways in atherosclerosis, revealing significant advancements in understanding the molecular mechanisms underlying inflammatory processes within arterial lesions. Notably, research has demonstrated that the modulation of microRNA‐146a (miR‐146a) expression impacts innate inflammation, effectively halts atherosclerosis progression, and enhances plaque stability by targeting interleukin‐1 receptor‐associated kinase (IRAK) and activating TNF receptor‐associated factor 6 (TRAF6), a signalling pathway involving toll‐like receptors (TLRs). Understanding the intricate mechanisms involved is crucial. This study provides a comprehensive analysis of the evidence and underlying mechanisms through which miR‐146a exerts its effects. Integrating these findings into clinical practice may herald a transformative era in managing atherosclerotic cardiovascular disease.

## 1 INTRODUCTION

Vascular diseases, such as ischemic heart disease and stroke, cause high mortality worldwide.^1^, ^2^ Most vascular diseases are primarily influenced by inflammation, such as through the secretion of cytokines, immune cell recruitment and cellular injury. Inflammation begins with the innate immune response to certain triggers, such as infection or cell injury, and this response leads to the release of innate immune cells. Several studies have suggested that granulocyte and platelet levels can represent innate immunity. The risk of vascular diseases and arterial calcification has increased due to innate immunity, marked by an elevated granulocyte count.^3^ This finding indicated the importance of innate immunity in the pathogenesis of vascular disease.

Atherosclerosis, the hardening of the arterial wall caused by forming lipid plaques (atheroma), is one of the most prevalent vascular diseases. Atheroma progression is affected by innate inflammation. Innate inflammation constitutes most of the pathophysiological process. One of the major risk factors for developing this condition is high blood cholesterol due to an imbalanced diet and low physical activity.^4^ Atherosclerosis may develop in various organs and cause different complications ranging from mild symptoms to death. Blockage of blood flow because of atherosclerosis in peripheral vascular disease may cause symptoms such as leg pain that can interfere with daily activities. Moreover, those that are formed in coronary arteries will lead to heart failure and death.^5^ Atherosclerosis occurs predominantly in bifurcations where the turbulence is high and more prone to endothelial damage.^6^

Up to now, atherosclerosis treatment strategies have mainly targeted blood cholesterol levels, predominantly low‐density lipoprotein (LDL) cholesterol. It can primarily be achieved by a group of drugs called statins that work by inhibiting the enzyme HMG‐CoA reductase, eventually decreasing cholesterol formation. Statins are beneficial for reducing plaque volume when used to manage coronary atherosclerosis. However, it could not alter plaque composition, so the risk of rupture remained.^7^ Plaques prone to rupture exhibit increased immune activity and inflammation.^8^ Thus, it is essential to resolve inflammation in atherosclerosis to prevent rupture and further complications. Therefore, targeting innate inflammation should be considered to better manage vascular disease.

The modulation of microRNAs (miRNAs) has provided promising evidence for treating innate inflammation in atherosclerosis. Some known miRNAs affecting the inflammatory process in atherosclerosis are miR‐146a, miR‐155, miR‐33 and miR‐21.^9^ In particular, the expression of miR‐146a has been found to be associated with reduced levels of inflammatory markers and more stable plaques. Therefore, this study aims to briefly overview the role of innate inflammation in atherogenesis and critical insights into studies of the potential therapeutic effect of miR‐146a on atherosclerosis.

## 2 INNATE INFLAMMATION IN ATHEROSCLEROSIS

The development of atherosclerosis begins with endothelial damage. Endothelial injury can be caused by smoking, hypercholesterolemia, hypertension and hyperglycaemia. Endothelial damage has increased permeability, which allows LDL to enter and reside below the arterial intimal layer and become oxidized. In addition to cholesterol deposition in the intimal layer, inflammation is also an important cause of plaque progression.^10^ Inflammation in atherosclerosis is referred to as sterile inflammation because it is stimulated by damage‐associated molecular patterns (DAMPs) from cell injury instead of pathogen‐associated molecular patterns (PAMPs) from infectious agents. Nonetheless, both stimuli produce the same inflammatory process.^11^ Innate inflammation represents a substantial part of the early development of atherosclerosis. This process involves stimuli to induce inflammation, monocytes, neutrophils, pattern recognition receptors and cytokines.^12^

### 2.1 Role of oxidized LDL in initiating innate inflammation

Oxidized LDL has numerous roles in initiating innate inflammation. It can activate endothelial cells to produce chemokines, bind to pattern recognition receptors, vascular smooth muscle cells and most importantly, monocyte recruitment, apoptosis and necroptosis in atherosclerosis.^13^ Oxidized LDL causes the endothelium to express surface adhesion molecules and attract monocytes, which activate the innate immune system. Monocyte transformation into macrophages is caused by macrophage colony‐stimulating factor. Macrophages then uptake oxidized LDL via scavenger receptors such as SR‐A and CD36. The absence of downregulation in the uptake of oxidized LDL causes the macrophage to be overloaded by lipid molecules and form foam cells.^12^, ^14^

Macrophages overloaded with oxidized LDL will undergo apoptosis mediated by caspase. In the early stages, apoptotic macrophages are cleared by efferocytosis. However, with an increasing amount of oxidized LDL and therefore macrophage apoptosis, efferocytosis becomes overwhelmed and ineffective in clearing these cells. In addition, caspases are ineffective, leading to another form of programmed cell death called necroptosis and the release of necrotic cell material. Together, these processes cause the formation of a necrotic core that permanently resides in the subendothelial layer.^15^, ^16^

### 2.2 Pattern recognition receptors

Pattern recognition receptors (PRRs) are structures in immune cells that are responsible for identifying DAMPs and initiating the innate immune response in atherosclerosis. One of the most important PRRs in the pathogenesis of atherosclerosis is the toll‐like receptor (TLR).^17^ TLR2 is one of the receptors that can bind to oxidized LDL in atherosclerosis, and deficiency of this receptor in mice has been shown to have a protective effect on atherosclerosis.^18^ TLR4 can also recognize oxidized LDL and initiate atherosclerotic plaque formation. It has been observed in mice that TLR4 deficiency leads to decreased monocyte recruitment and atherosclerotic progression.^19^ The TLR3 signalling pathway differs from other TLRs because it is independent of myeloid differentiation primary‐response protein 88 (MyD88). However, TLR3 is also important for the release of proinflammatory cytokines in atherosclerosis, which causes plaque instability.^20^

Another type of PRR called nucleotide‐binding oligomerization domain (NOD)‐like receptors (NLRs) has also been shown to be involved in atherosclerosis progression. NLR activation by DAMPs and oxidized LDL induces the formation of several inflammasome types. Among these inflammasomes, the NLR family pyrin domain containing 3 (NLRP3) inflammasome is the most crucial type involved in innate inflammation in atherosclerosis because it is highly expressed in monocytes and macrophages.^21^ The generation of the NLRP3 inflammasome causes the release of IL‐1β, which increases vascular smooth muscle proliferation, further monocyte recruitment and promotes the production of cytokines and chemokines that threaten plaque stability. The NLRP3 inflammasome also causes proptosis of endothelial cells, macrophages and smooth muscle cells via the activation of caspase 1. The death of endothelial cells exacerbates the influx of LDL and monocytes into the subintimal layer, while the death of macrophages and smooth muscle cells leads to a greater proportion of necrotic cores.^22^

### 2.3 Inflammatory cytokines and plaque instability

Atherosclerotic plaques can rupture due to shear stress. The rupture of the plaque will activate the coagulation cascade and cause thrombosis. Thrombus can become dislodged and cause emboli in smaller arteries, or the size can be large enough to obstruct the whole arterial lumen. Both can cause diseases such as acute myocardial infarction and ischemic stroke. Thus, it is important to maintain the stability of atherosclerotic plaques.^23^ Several features can distinguish vulnerable plaques from stable plaques. Vulnerable plaque has a hallmark that consists of a large necrotic core, a high lipid composition, and an inflamed thin fibrous cap called a thin cap fibroatheroma.^24^ These features result from the release of inflammatory mediators such as IL‐6, TNF‐α and MMP that are triggered by LDL binding to macrophages and NLRP3 inflammasome activation.^8^

## 3 ANTI‐INFLAMMATORY EFFECT OF miR‐146A ON ATHEROSCLEROSIS

MiRNAs are 22‐nucleotide noncoding RNAs involved in regulating gene expression and physiological processes such as differentiation, development and function of cells in the body.^25^ The effects of miRNAs on inflammatory responses were shown by genetic disruption of their biogenesis in macrophages. One of the most representative candidates for thrombo‐inflammation is miR‐146a.^26^ Recent studies have indicated that miR‐146a is involved in inflammatory diseases and contributes to the initiation and progression of atherosclerosis.^27^, ^28^, ^29^ It has been identified as a suppressor of proinflammatory adaptor proteins factor κ light chain enhancer of activated B cells signalling pathway in endothelial and bone marrow (BM) derived cells.^30^

**FIGURE 1:**
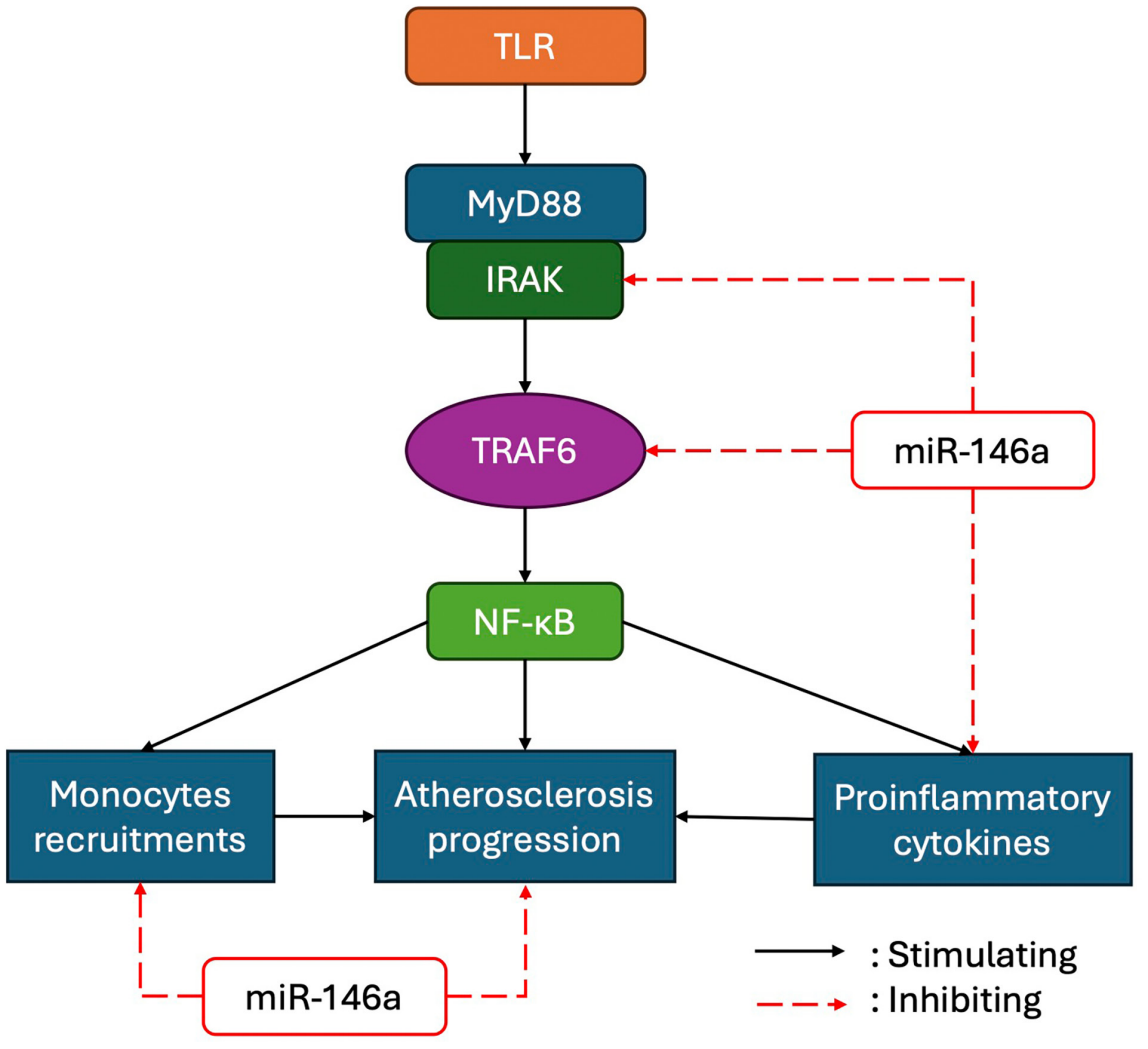
The role of miR‐146a as a molecular brake of inflammation in atherosclerosis. miR‐146a exerts an inhibitory role in the TLR signalling pathway by suppressing the activation of IRAK and TRAF6. Additionally, it negatively regulates proinflammatory cytokine production, monocyte recruitment, and the progression of atherosclerosis. IRAK, interleukin‐1 receptor‐associated kinase; MyD88, myeloid differentiation primary‐response protein 88; NF‐KB, nuclear factor kappa B; TLR, toll‐like receptors; TRAF6, tumour necrosis factor receptor‐associated factor 6.

The mechanism underlying the role of miR‐146a as a critical molecular brake of inflammation involves targeting the TLR signalling pathway (Figure 1).^31^, ^32^ The binding of TLRs to oxidized LDL can activate the MyD88 signalling pathway. The death domain of MyD88 then binds to interleukin‐1 receptor‐associated kinase (IRAK) and activates TNF receptor‐associated factor 6 (TRAF6). TRAF6 activation eventually leads to the activation of nuclear factor kappa B (NF‐κB), which causes macrophage apoptosis and the production of proinflammatory cytokines.^17^, ^33^, ^34^ IRAK and TRAF6 are targets of miR‐146a, which mediates the suppressive effect of miR‐146a on innate inflammation in atherosclerosis.^32^ Moreover, miR‐146a also has a role in preventing endothelial dysfunction by inhibiting endothelial activation with increasing nitrite oxide synthase (eNOS) expression and reactive oxygen species (ROS) inhibition via NADPH Oxidase 4 (NOX4) pathway.^28^, ^35^

### 3.1 In vitro study

An in vitro study by Yang et al. revealed an interesting relationship between miR‐146a and atherosclerosis. There was a statistically significant downregulation of miR‐146a in macrophages with oxidized LDL, and the administration of the miR‐146a mimic caused a significant decrease in the concentration of intracellular LDL in macrophages. Another important finding was the reduction of cytokines level because of the inactivation of TLR4 by miR‐146a mimic transfection. It was speculated that TLR4 is a target through which miR‐146a reduces atherosclerosis progression.^31^ However, the sample cell of this study was minimal, so interpretation should be made with caution. Furthermore, THP‐1 cells in this study might not represent the actual conditions. Thus, the experiment should be replicated in primary human monocytes for comparison to strengthen the significance of the results of this study.

### 3.2 In vivo studies

Chu et al. investigated the protective effect of miR‐146a in a mouse model of atherosclerosis and RAW264.7 macrophages. One of their results showed that miR‐146a inhibits atherosclerosis progression by decreasing foam cell formation, which is in concordance with the findings of a previous in vitro study by Yang et al.^31^, ^32^ Moreover, the most important finding in their research was the protective effect of miR‐146a against plaque instability. Vulnerable plaque features include a high proportion of lipids and apoptotic macrophages and a high level of proinflammatory cytokines. Histopathological staining using haematoxylin‐eosin in the atherosclerotic aortas of the mice showed that injection of the miR‐146a agonist significantly reduced the area of lipids and macrophages. This might be explained by the in vitro finding that RAW264.7 macrophages expressing miR‐146a showed significantly lower lipid uptake and apoptosis rates.^32^

Xiao et al. assessed the anti‐inflammation and oxidative stress effects of miR‐146a in vivo. The miR‐146a decreased the inflammatory response by reducing CD68‐labelled macrophage infiltration, levels of TNFα and IL‐1. The miR‐146a mimic also reduced the ROS level in ischemic/reperfusion (I/R) injured mouse hearts, increased mitochondrial antioxidant manganese superoxide dismutase (MnSOD) activity and diminished nicotinamide adenine dinucleotide phosphate (NADPH) activity.^28^

Cheng et al. showed that in the absence of miR‐146a in the BM‐derived cells mouse model, increasing the production of proinflammatory cytokines while reducing circulating proatherogenic leukocytes resulted in decreased atherosclerosis. This study also proved that miR‐146a in BM‐derived cells protects against high cholesterol diet‐induced haematopoietic progenitor cell exhaustion in the BM and prevents extramedullary haematopoiesis and splenomegaly. Furthermore, the level of circulating very low‐density lipoprotein (VLDL) is significantly decreased in mice lacking miR‐146a in the BM. This is also accompanied by enhanced inflammation in the liver and dysregulation of a newly identified miR‐146a target gene, sortilin 1.^30^

The anti‐inflammatory property of apolipoprotein E (apoE) could protect against atherosclerosis by regulating cellular microRNA levels in leukocytes. An in vivo study by Li et al. revealed that cellular apoE expression suppresses nuclear factor‐κB–mediated inflammation and atherosclerosis by enhancing miR‐146a levels in monocytes and macrophages. This study demonstrated that even a small amount of apoE expression in macrophages and monocytes of hippomorphic apoE mice led to increased miR‐146a levels and inhibited macrophage proinflammatory responses, monocytosis and atherosclerosis in hyperlipidaemia.^36^ Also, it has been reported that the delivery of miR‐146a using an E‐selectin‐targeting multistage vector to inflamed endothelium covering atherosclerotic plaques decreases plaque size and macrophage infiltration in apoE mice.^37^

### 3.3 Clinical studies

In 2021, Pereira‐da‐Silva et al. investigated whether metabolic, inflammatory and epigenetic miRNA markers are associated with TNF‐α expression in 24 patients with stable coronary artery disease (SCAD). They found that miR‐146a expression levels were negatively correlated with TNF‐α levels. However, it should be noted that the sample size of this study was small, and the patients were mostly male. Additionally, a causal relationship between miR‐146a and TNF‐α cannot be established based on the results of this study.^29^

Despite the presumed anti‐inflammatory effect of miR‐146a, a study by Zhu et al. showed that there was significantly greater expression of miR‐146a in the circulating mononuclear cells of patients with vulnerable plaques than in those of patients with stable plaques.^27^ However, some points should be considered before interpreting this study. First, the study was not powered to determine associations. Second, detecting miR‐146a expression in circulating mononuclear cells may not be relevant to atherosclerosis and cannot be compared to other studies using macrophages from plaques or activated by oxidized LDL.

### 3.4 Future direction of miR‐146a

Even though miR‐146a has a relationship with atherosclerosis, it cannot be considered specific, as it has been shown to be involved in various diseases such as malaria and its complications, cancer and other conditions due to its ability to influence immune regulatory genes and targeting innate inflammation.^38^, ^39^, ^40^ Therefore, therapeutic strategy using miR‐146a must be carefully considered to maintain its specificity, minimizing unintended effects on other conditions. Milk exosomes (ME) can be loaded with miR‐146a and target specific cells, such as myocardial injury sites. Besides, miRNAs can be conjugated with peptides or antibodies that bind to specific cell surface markers to deliver the desired cell type precisely. These methods can protect miRNAs from ribonuclease degradation and enhance their delivery specificity. Meng et al. showed that using MEs‐miR‐146a in oral administration decreased myocardial tissue apoptosis and the expression of inflammatory factors in rat models. It also improved cardiac function after myocardial ischemia–reperfusion injury (MIRI). By using intravenous injection, it may enhance the targeting of the heart, improve cardiac function, reduce myocardial tissue apoptosis and suppress inflammation.^41^, ^42^

Aside from miR‐146a, other microRNAs are associated with plaque instability and cardiovascular outcomes. MiR‐125a helps decrease the lipid uptake in macrophages and modulates extracellular vascular endothelial growth factor (VEGF) by manipulating macrophage soluble VEGF receptor‐1 (sVEGFR1) production. Taken together with miR‐146a, this combination has therapeutic potential in many diseases, notably in the early development of atherosclerosis.^35^, ^43^ The expression levels of miR‐122‐5p, miR‐1‐3p and miR‐16‐5p during acute ischemic events may be potential biomarkers for predicting the risk of secondary cardiovascular events, such as myocardial infarction and ischemic stroke. MiR‐122 was found to be associated with lipid metabolism and lipid uptake, which might show independent associations with cardiovascular death, future episodes of stroke and myocardial infarction. MiR‐1‐3p and miR‐16‐5p may affect pathways related to inflammation, apoptosis and cellular stress responses. Dysregulation of miR‐1‐3p and miR‐16‐5p also contributes to adverse cardiac remodelling. In addition, miR‐16‐5p has been shown to influence endothelial function and smooth muscle cell proliferation, which affect plaque stability and the risk of acute cardiovascular events.^44^ Utilizing miRNAs for diagnostic and therapeutic purposes may herald a transformative era in managing cardiovascular diseases.

## 4 CONCLUSION

Most studies have shown promising results in increasing plaque stability by targeting innate inflammation in atherosclerosis. However, further studies are still needed to determine the effect of these therapies on humans, their side effects, mode of administration and many other conditions. Targeting innate inflammation combined with existing statin therapy is expected to reduce the burden of morbidity and mortality from atherosclerotic plaques.

## AUTHOR CONTRIBUTIONS

**Azizah Puspitasari Ardinal:** Conceptualization (lead); investigation (equal); writing – original draft (equal); writing – review and editing (equal). **Alice Valeria Wiyono:** Conceptualization (equal); investigation (equal); writing – original draft (equal); writing – review and editing (equal). **Reza Ishak Estiko:** Conceptualization (equal); investigation (equal); writing – original draft (equal); writing – review and editing (equal).

## FUNDING INFORMATION

None to be declared.

## CONFLICT OF INTEREST STATEMENT

The author declares that no competing interest exists related to the content of the review.

## Data Availability

This paper is exempt from data sharing.
